# A Comprehensive Assessment of Bedtime Routines and Strategies to Aid Sleep Onset in College Students: A Web-Based Survey

**DOI:** 10.3390/clockssleep6030031

**Published:** 2024-08-29

**Authors:** Debora Meneo, Sara Curati, Paolo Maria Russo, Monica Martoni, Francesca Gelfo, Chiara Baglioni

**Affiliations:** 1 Department of Human Sciences, Guglielmo Marconi University, Via Plinio, 44, 00193 Rome, Italy; d.meneo@unimarconi.it (D.M.);; 2 Department of Medical and Surgical Sciences, University of Bologna, 40138 Bologna, Italy; 3 IRCCS Fondazione Santa Lucia, 00179 Rome, Italy; 4 Department of Psychiatry and Psychotherapy, Medical Center—University of Freiburg, Faculty of Medicine, University of Freiburg, 79104 Freiburg, Germany

**Keywords:** pre-sleep behaviours, bedtime routines, sleep aid, insomnia, sleep health, college students

## Abstract

College students often experience sleep–wake alterations. Different factors can contribute to insomnia symptoms in this population. The present study aims at investigating pre-sleep behaviours and strategies used to aid sleep onset in young college students and their association with insomnia symptoms. A total of 548 Italian college students (mean age = 23.48 years, range = 19–30 years, 80.5% female) completed a web-based survey on pre-sleep behaviours and sleep-onset facilitators, insomnia symptoms and sleep hygiene, anxiety and depression, and coping strategies. The use of electronic devices at bedtime and as a sleep-onset facilitator was predominant. Students using specific behaviours as sleep-onset facilitators were characterised by more psychological difficulties and poorer sleep. In multivariable linear regression analysis, the frequency of using medications and melatonin, regardless of motivations, was associated with higher insomnia symptoms. The use of specific sleep-onset facilitators positively correlated with the severity of insomnia symptoms. Many students engage in behaviours that are considered sleep-interfering and that are often employed in an attempt to facilitate sleep onset without benefits. Overall, the motivational factors behind pre-sleep behaviours need to be addressed in preventive programs targeting young college students.

## 1. Introduction

The sleep–wake cycle is regulated by two processes concurring in consolidating sleep and wake periods: a homeostatic process, representing sleep pressure, which increases during waking hours, and a circadian process, which synchronises sleep–wake periods with the external light–dark cycle [1]. The optimal moment for sleep initiation is at a point in which the homeostatic pressure and the circadian propensity for sleep are maximum. In this point of the 24 h cycle, there is a natural cognitive and somatic deactivation facilitating sleep onset [2]. People without sleep difficulties make little active effort to sleep, and a range of internal (e.g., sleepiness) and external (e.g., the bedroom environment) conditioned cues facilitate the passage between wakefulness and sleep [2]. In childhood, this automated deactivation is linked to bedtime routines, defined as predictable and repetitive behaviours occurring in the hours before lights out [3]. Bedtime routines can help create a suitable sleep environment and a way of winding down in the pre-sleep period, allowing for deactivation and facilitating sleep. Mindell and Williamson [4] underlined that the engagement in healthy pre-sleep activities (e.g., reading and listening to music) is important for sleep outcomes in children. The inability to deactivate at sleep onset is a characteristic of chronic insomnia disorder in adults, with hyperarousal being a 24 h feature of the disorder [5]. Insomnia disorder is a prevalent sleep–wake disorder (approximately 10% of the adult population), and it is characterised by difficulties in sleep initiation and/or maintenance and subjective dissatisfaction with sleep, coupled with daytime dysfunctions (e.g., fatigue, low mood, difficulties in concentration) [6]. Insomnia is associated with negative consequences both on the individual level (i.e., distress) and on the societal level (e.g., reduced productivity, increased costs on the healthcare system). Healthy bedtime routines can, thus, be beneficial also for adults struggling to deactivate at bedtime, such as those suffering from insomnia disorder or experiencing insomnia symptoms. On the other hand, poor sleep hygiene practices, including unhelpful pre-sleep behaviours, are a target of the Cognitive Behavioural Therapy for Insomnia (CBT-I) [7]. Pre-sleep behaviours may be essentially different across ages. For instance, bedtime routines in young children are often set by caregivers, while adults have much more control over their behavioural choices at bedtime. It is, thus, important to have a deeper understanding of which pre-sleep behaviours are mostly performed in different populations and how they are associated with sleep.

College students may be particularly at risk of employing harmful pre-sleep behaviours [8]. Young college students often suffer from inadequate sleep [9]. Specifically, college students are more vulnerable to developing a sleep disorder, including insomnia [9]. Students experiencing insomnia are at a higher risk of psychological problems, substance abuse, and reduced academic performance and quality of life [10,11]. Factors associated with poor sleep in college students include poor sleep hygiene (practices and environmental conditions that can interfere with the homeostatic and/or circadian regulation of sleep), symptoms of anxiety and depression, and circadian preference (eveningness) [12,13,14]. College years are a demanding life period in which young adults face the challenges of entering the adult life together with specific stressors, such as academic assignments and tests, new environments, and changes in social life and family relations [15,16]. All of these factors can contribute to increasing levels of anxiety and depression, which are strongly associated with insomnia symptoms (e.g., [17,18]). Moreover, recently, there has been an increasing interest in the psychological factors associated with sleep difficulties and which ones can increase vulnerability to insomnia disorder, such as the ability to cope with stress [19]. Coping refers to the efforts to regulate emotions, behaviours, thoughts, physiological responses, and the environment in stressful situations [20]. Although the effectiveness of a coping strategy may be dependent on the context, avoidant and emotion-focused coping are considered less adaptive than problem-focused coping [21,22]. Maladaptive coping strategies have been associated with sleep quality, i.e., a less-effective coping style may increase the impact of life stressors on sleep [23]. Specifically, maladaptive coping styles may lead to increased perceived negative impacts of stressors, thus exacerbating the stress response; this, in turn, can contribute to difficulties in regulating arousal in patients with insomnia disorder (i.e., physiological arousal, excessive worry) [19].

In the context of increased vulnerability of college students to sleep difficulties, there is a need to understand the behavioural factors that can facilitate or inhibit the natural deactivation at bedtime and, thus, contribute to or maintain sleep problems in this population. These behaviours can then be targeted in interventive and preventive approaches. The literature has focused on poor sleep hygiene practices in college students, which is considered one major contributor to sleep difficulties [24]. Sleep hygiene practices are routinely assessed using the Sleep Hygiene Index [25], which includes items in the bedroom environment, the use of substances in the 4 h preceding bedtime, behaviours that enhance arousal close to bedtime, and daytime naps. While the assessment of sleep hygiene is important in college students, bedtime behaviours extend beyond sleep hygiene practice. For instance, assessing sleep hygiene practice gives little specific knowledge about the individual’s intended use and the use of specific behaviours close to bedtime and, thus, the specific contribution of such behaviours (e.g., listening to music, hygiene-related behaviours, consumption of different beverages). The literature has particularly focused on the use of substances and electronic devices at bedtime in young adults [26,27]. The use of sedating or activating substances, including alcohol, cannabis, nicotine, and caffeine, can alter sleep architecture [28]. The exposure to bright light, such as those of electronic devices, close to bedtime can shift the circadian rhythm and delay sleep onset and, thus, alter the circadian regulation of sleep [29]. Because behavioural choices are dependent on motivations, beliefs, and cultural factors, attempts to directly change these behaviours can be challenging without a comprehension of motivational factors. Considering motivations can lead to more effective preventive and interventive programs. However, research on this topic is scarce. The bedtime use of electronic devices and substances can be reinforced in young adults by coping responses to stress and sleep problems [26,30]. Young adults are increasingly using substances, particularly alcohol and cannabis, to facilitate sleep, despite the negative effect of substance use on sleep [31]. The use of substances to facilitate sleep is not associated with better sleep outcomes; indeed, while substance use is associated with impairments in different sleep health dimensions, their use to aid sleep is associated with more negative consequences on sleep health [32]. In fact, the use of substances to self-medicate sleep problems is associated with increasing sleep difficulties and, thus, increased use of the substance, leading to a vicious circle [33]. Moreover, the self-medication of sleep difficulties has been also associated with more psychological difficulties, including higher anxiety and depression [26].

Recently, attention has been paid to the motivations behind other common bedtime behaviours, such as social media use. Indeed, technology before bedtime can be used as a way to tone down cognitive activity in the evening and to facilitate sleep onset [34,35]. In a sample of adolescents from Australia, more than half used technology, especially social media apps, as a distraction from unpleasant thoughts in the evening, and this was mostly endorsed by adolescents with sleep problems [34]. However, different behaviours can be used to facilitate sleep onset beyond substance and technology use. Indeed, in the general population, approximately one-third of adults employ some strategies to fall asleep in the form of common bedtime routines, including reading and listening to music [36,37]. Overall, there are indications that pre-sleep behaviours can be sustained by motivations to facilitate sleep. However, there is a lack of investigations on different behaviours, the characteristics of those using behaviours to facilitate sleep, and how this motivation can be associated with sleep difficulties. The association between use as a sleep aid and insomnia may be accounted for by different mechanisms. Those with insomnia may be more prone to cognitive and behavioural efforts to control sleep processes, i.e., they may more likely perform some behaviours with the intention to facilitate sleep onset [1]. For instance, reading before bed can be a pleasurable activity that can naturally lead to relaxation; consequently, the person would try to stay awake as long as possible to continue reading, with the paradoxical effect of facilitating sleep onset. On the contrary, people with chronic insomnia can try to read with the intention to seek sleep, resulting in frustration, negative emotions, and more arousal [2]. This active effort to facilitate sleep can increase sleep-interfering arousal at bedtime [1]. Another mechanism can be the need to tone down negative emotions and thoughts associated with sleep-onset difficulties [38]. The model of insomnia developed by Harvey [38] focuses on the negative cognitive activity that perpetuates sleep difficulties, and which is aggravated by active efforts to suppress it in the form of safety behaviours. These are behaviours, such as drinking alcohol, that the person employs to cope with sleep difficulties by attempting to reduce the negative cognitive activity associated with sleep onset [39].

The evidence so far has focused on single pre-sleep behaviours (substance use and electronic device use) considered as potentially detrimental to sleep in emerging adults. There is a lack of comprehensive assessments of bedtime routines in young adults, the strategies used to aid sleep onset (i.e., sleep-onset facilitators), and the psychological factors associated with the use of sleep-onset facilitators. While sleep hygiene practices are important in this population, different pre-sleep behaviours not included in the standard definition of sleep hygiene can also be associated with sleep health. Moreover, there is a need to better understand the motivational factors of pre-sleep behaviours to plan effective programs aimed at behavioural change. Indeed, intentions are the antecedents of behaviours, and, thus, to change harmful habits, it is pivotal to understand, among other factors, the intention preceding them [40]. As reviewed above, pre-sleep behaviours can be performed to control sleep or to reduce negative emotions and thoughts. In addition, most of the literature here reviewed is based on studies performed in college students from the United States. It is unclear how cultural factors can influence pre-sleep behaviours, bedtime routines, and their association with sleep health. Cross-cultural analysis in infant, children, and adolescents showed that bedtime routines and habits change between counties (including Asia, Europe, North America, Australia, and the Middle East region) [41]. Moreover, sleep patterns and beliefs and attitude about sleep also show cultural differences in college students (e.g., between Japan and Canada) [42]. More data are needed in different cultural contexts to have a clearer picture of pre-sleep behaviours in college students. Indeed, to our knowledge, no studies have investigated in detail pre-sleep behaviours and the use of different sleep-onset facilitators in young college students.

### Objectives and Hypotheses

Based on the above, the present explorative study on college students aims at the following:

1. Collecting information on specific pre-sleep behaviours and strategies used as sleep-onset facilitators;
2. Characterising those using specific sleep-onset facilitators in terms of psychological variables (symptoms of anxiety and depression) and sleep habits;
3. Investigating the association between specific pre-sleep behaviours and insomnia symptoms;
4. Investigating the association between the use of sleep-onset facilitators and insomnia symptoms.

Considering the literature so far, we tested the following hypothesis concerning objective 3 and 4:

- We expected that pre-sleep behaviours would significantly contribute to insomnia symptoms; in particular, we expected that a higher frequency of pre-bedtime use of electronic devices and substances would be associated with higher insomnia symptoms, while the use of relaxing behaviours, such as reading and listening to music, would be associated with lower insomnia symptoms. Based on the literature, we could expect that other significant contributors to higher insomnia symptoms would be levels of anxiety and depression, less-effective coping strategies (avoidant and emotion-focused coping), poorer sleep hygiene, and more evening circadian preference.
- We expected that the use of some common pre-sleep behaviours, such as substance use, as sleep-onset facilitators would be associated with higher insomnia symptoms.

## 2. Results

### 2.1. Sample Characteristics

A total of 548 college students (80.5% females) aged 19–30 years (M_age_ = 23.48; SD = 2.77) participated in this study. Table 1 reports the demographic characteristics and psychological status of the sample (academic variables are available as Supplementary Table S1).

The students came from universities across all Italian macroregions, with a slightly higher prevalence from South Italy (35.8%) (Italian macroregions are first-level NUTS (Nomenclature d’Unités Territoriales Statistiques, Classification of Territorial Units for Statistics) of the European Union. NUTS is a geocode for administrative divisions of European countries used for statistical purposes). The majority were non-working students (75.2%), in a relationship (55.6%), and living with the family of origin (64.6%). A total of 12.8% were in their first year of university, 20.8% were in the last years of university, while 15.7% were outside the prescribed time. At the time of the survey, 10.2% of the students had finished all mandatory exams. Exam proximity for the remaining students was approximately evenly distributed (see Supplementary Table S1 for details).

Table 1 shows the mean scores and standard deviations of sleep and psychological measures. Average scores were above the cut-off for subclinical or threshold symptoms for insomnia (10.53 ± 5.81), anxiety (9.23 ± 4.00), and depression (8.29 ± 3.97). Moderate to severe symptoms of insomnia (ISI > 14), anxiety, and depression (HADS-Subscales > 10) were reported, respectively, by 26.1%, 38.1%, and 31.8% of students. Most of the sample (85.4%) reported good sleep hygiene practices (SHI ≤ 26). The distribution of circadian typologies indicated a higher prevalence of evening-types (21.9%) compared to morning-types (12.0%); evening-types were more prevalent in our sample compared with previous reports on Italian young adults (e.g., 13.56%) [43].

Regarding sleep patterns, more students reported an earlier bedtime and wake time during exams compared to non-exam periods, particularly during weekends (Supplementary Table S2).

### 2.2. Pre-Sleep Behaviours and Strategies Used as Sleep-Onset Facilitators

Electronic device use stands out as the most common bedtime routine, with 94% and 76% of students spending time on social media and watching TV shows as a pre-sleep behaviour at least three days per week (see Supplementary Table S3). Other bedtime routines were less frequently endorsed and included taking a hot bath or shower (41%) and listening to music (41%). The use of substances and medications was also habitual for a small percentage of students, with alcohol use (17%), smoking cigarettes or e-cigarettes (25%), and taking melatonin (16%) being the most endorsed. On average, the students spent time on social media and watching TV shows before bed for 5.75 ± 1.71 and 4.56 ± 2.42 days, respectively. Taking a bath and listening to music were reported twice a week on average and reading for 1.71 ± 2.14 days per week. All other behaviours were less frequent on average, with a mean frequency of less than 3 days a week (see Supplementary Table S3).

Table 2 reports the number and percentage of students reporting each pre-sleep behaviour at least once a week, the use to facilitate sleep onset, and the perceived effectiveness in aiding sleep. Overall, 70.4% of students reported using at least one strategy to fall asleep more easily. The most common bedtime routines were also the most endorsed sleep-onset facilitators: a total of 51.3% reported watching TV shows, and 43.8% reported spending time on social media to facilitate sleep onset. Pre-sleep behaviours were rarely perceived as effective in aiding sleep, with the most effective being watching TV shows (reported as effective by 29.9% of the sample).

### 2.3. Characteristics of Students Using Sleep-Onset Facilitators

Independent *t*-tests were performed to compare the students reporting use of behaviours as sleep-onset facilitators and students reporting use for other motivations on insomnia (ISI), anxiety and depressive (HADS) symptoms, and coping strategies (BRIEF-COPE). To enhance the power of the analyses, we firstly created the following groups of behaviours based on reported use to facilitate sleep onset: “relaxing behaviours” comprised putatively of de-arousing behaviours based on previous literature (at least one of the following was used to facilitate sleep onset: taking a hot bath or shower, listening to music, reading, drinking herbal tea, doing meditation, doing yoga [44,45,46]; *n* = 303); “substance use” (at least one of the following: alcohol, cannabis, smoking cigarettes, other substances; *n* = 84), “medication use” (at least one of the following: prescribed sleep medications, non-prescribed sleep medications, melatonin, antihistamine; *n* = 114); and “electronic devices use” (at least one of the following: spending time on social media, watching TV shows; *n* = 356).

Table 3 reports the results of the *t*-test. Those endorsing relaxing behaviours as sleep-onset facilitators (*n* = 303) reported significantly higher insomnia symptoms (11.39 ± 5.77) than those not reporting the motivation of sleep-onset facilitation (*n* = 188; 9.42 ± 5.84), *t*(489) = 3.65, *p* < 0.01). The students using substances (*n* = 84 vs. *n* = 203) and medication (*n* = 114 vs. *n* = 83) as sleep-onset facilitators showed a similar profile characterised by significantly higher insomnia symptoms, poorer sleep hygiene, and higher anxiety symptoms (*p* < 0.05). Specifically, those using any medication to aid sleep onset reported higher scores on ISI (14.12 ± 5.31 vs. 10.52 ± 5.69, *t*(195) = 4.51, *p* < 0.0001), SHI (20.81 ± 7.66 vs. 18.17 ± 7.50, *t*(195) = 2.42 *p* < 0.05), and HADS-Anxiety (10.68 ± 3.85 vs. 9.10 ± 4.05, *t*(195) = 2.77, *p* < 0.05) compared to use for other motivations. Those reporting the use of any substance to aid sleep onset reported higher scores on ISI (13.26 ± 5.86 vs. 10.49 ± 5.52, *t*(285) = 3.7 *p* < 0.01), SHI (22.68 ± 7.74 vs. 19.23 ± 6.93, *t*(285) = 3.54 *p* < 0.05), and HADS-Anxiety (10.25 ± 4.06 vs. 9.15 ± 3.81, *t*(285) = 2.13, *p* < 0.05) compared to use for other motivations.

To further explore the profile of the students using specific pre-sleep behaviours as sleep-onset facilitators, we performed separate analyses for the most endorsed pre-sleep behaviours with the motivation to facilitate sleep onset: social media use (*n* = 240), watching TV show on any device (*n* = 281), listening to music (*n* = 133), and taking a hot shower or bath (*n* = 112). The *t*-test results showed a statistically significant result only for listening to music: those endorsing use as a sleep-onset facilitator reported significantly higher insomnia symptoms (12.17 ± 6.13) compared to those not reporting this motivation (9.79 ± 5.62), *t*(300) = 3.48, *p* = 0.0006.

### 2.4. Contribution of Pre-Sleep Behaviours to the Severity of Insomnia Symptoms

The correlations between all continuous study variables are reported in Table 4. Among the pre-sleep behaviours, the frequency of use of melatonin (r = 0.27) and prescribed medication (r = 0.23) were more strongly correlated with insomnia symptoms (*p* < 0.0001).

The result of the stepwise multiple linear regression model predicting insomnia symptoms is reported in Table 5 and displayed in Supplementary Figure S1. We included, in the first step, all the variables identified by the literature that could be associated with insomnia symptoms, including age, sex, exam proximity and year of university, coping strategies (avoidant, emotion-focused, problem-focused), sleep hygiene, anxiety and depression, and chronotype. Among the independent variables, we included, in the first steps, the frequency of all pre-sleep behaviours assessed, regardless of motivations reported to perform that behaviour (i.e., sleep-onset facilitation). The final stepwise model included, among pre-sleep behaviours, the following behaviours that were significant in the model: meditation, use of prescribed medications, use of melatonin, social media use, and drinking hot cocoa.

The regression equation for the total score of the ISI was significant (R^2^ = 0.4099; *p* < 0.0001). As expected, higher anxiety and depressive symptoms, poorer sleep hygiene, and use of avoidant coping were significant predictors of insomnia symptoms. Among pre-sleep behaviours, the larger effects were for the frequency of using prescribed sleep medications and melatonin. A higher frequency of using social media and drinking hot cocoa before bedtime were associated with lower insomnia symptoms.

### 2.5. Associations between Sleep-Onset Facilitators and Insomnia Symptoms

The results of the correlations between the behaviours used as sleep-onset facilitators and insomnia symptoms are reported in Table 6. We considered both the total score of the ISI and the score of the first item, which specifically assess sleep-onset difficulties. Significant correlations were found between insomnia symptoms and the use, as sleep-onset facilitators, of alcohol, cigarette smoking, prescribed medications, melatonin, antihistamines, and listening to music. No significant negative correlations were found, indicating that no sleep-onset facilitator was associated with lower insomnia symptoms.

## 3. Discussion

This is, to our knowledge, the first comprehensive report of young adults’ pre-sleep behaviours and use to facilitate sleep onset. We collected information on the most common pre-sleep behaviours and the types of strategies used to facilitate sleep onset in college students. We also characterised those using specific sleep-onset facilitators in terms of psychological variables and sleep health. The associations between pre-sleep behaviours and sleep-onset facilitators with insomnia symptoms were tested. Our study included different behaviours, from the ones mostly investigated in relation to sleep (e.g., substance use, electronic device use) to those considered as positive pre-sleep behaviours (e.g., reading, meditation). In our sample of young college students (19–30 years), we found a high frequency of electronic device use during the hour preceding bedtime and a high endorsement of any strategy to facilitate sleep onset. From the *t*-tests analysis, the students employing specific behaviours as sleep-onset facilitators showed worse psychological and sleep health profile than those employing behaviours for other reasons. The use of avoidant coping strategies, poorer sleep hygiene, higher anxiety and depressive symptoms, and a higher frequency of using melatonin and prescribed sleep medications before bedtime were significant predictors of higher insomnia symptoms. Correlation analysis showed that the use of sleep-onset facilitators was associated with higher insomnia symptoms and that no specific behaviours used as sleep-onset facilitators were associated with a clear benefit on sleep. Overall, the results highlight the widespread use of pre-sleep behaviours, which can negatively impact sleep, and a high proportion of college students use behaviours to facilitate sleep onset without benefits for sleep.

The present study offers a comprehensive assessment of bedtime behaviours and use of sleep-onset facilitators in young college students. The bedtime routine mostly endorsed in our sample of Italian college students was the use of electronic devices. This is in line with previous literature in other countries underlining the widespread use of electronic devices at bedtime from childhood to early adulthood [47]. In our study, we did not find an association between the frequency of pre-bedtime electronic device use and sleep impairment. The use of electronic devices at bedtime is generally discouraged for their negative impact on the circadian rhythm and for reduction in sleep duration due to bedtime procrastination [47]. However, electronic device use at bedtime is not always found to be associated with strong negative effects on sleep in adults. In a recent study, Ellithorpe et al. [48] suggested that the effect of electronic devices at bedtime may not be as detrimental to sleep, as in some situations (e.g., social isolation, high stress), they may have a positive effect on psychological health. The literature identified some factors that may moderate the association between technology use and sleep, such as the timing of use, screentime, and level of engagement with digital contents [48,49]. Moreover, the non-significant association between some pre-sleep behaviours and insomnia symptoms does not exclude their role on other dimensions of sleep health. Following the definition offered by Buysse [50], sleep health is a multidimensional construct comprising sleep duration, satisfaction, timing, efficiency, and daytime vigilance. In children, a sixth dimension of sleep-related behaviours has been proposed by Meltzer et al. [51]. In young adults, bedtime behaviours may be associated with specific sleep health dimensions (e.g., sleep duration and timing) [52]. For instance, social media use can lead to a delay in bedtime and, thus, to reduced sleep duration when waketime needs to be early [53]. There is, thus, a need to investigate the role of pre-sleep behaviours in association with other dimensions of sleep health in adults.

The rate of use of sleep-onset facilitators in our samples was higher than the rate of sleep aid use previously reported in the general population. Bjorvatn et al. [36] found that 34.3% of Norwegian adults between 18 and 70+ years of age reported any active strategy to aid sleep. Morin et al. [37] also reported that between 20.7% and 32.5% of Canadian adults (18–91 years old) used common pre-sleep behaviours (relaxation, listening to music, and reading) as strategies to promote sleep. To our knowledge, the present study is the first to directly investigate the use of different behaviours as sleep aids in a sample of college students classified as young adults. The higher comprehensiveness of the strategies investigated can, thus, explain the higher rate of sleep aid use. However, it should be further investigated how this difference can be influenced by cultural factors, such as different beliefs about the effect of some behaviours on sleep. Regarding single bedtime behaviours, the rate of substance use to aid sleep in our sample was comparable to previous reports on U.S. college students (7% to 15%) [26]. In our sample, the use of electronic devices as sleep aids was endorsed by half of all students, against a prevalence found in adults between 13.4% [36] and 31.2% [54]. Future studies could compare the prevalence of using social media to facilitate sleep onset between young adults and older adults in order to shed light on age differences.

The students using substances and medications as sleep-onset facilitators were characterised by higher symptoms of anxiety and insomnia symptoms. This is in line with the literature on sleep aid use, indicating a multimorbidity profile that can increase the risk of adverse outcomes [26,33] and, thus, a possible convergence in self-medication of sleep and psychological difficulties. This is particularly alarming, considering the high rate of insomnia and psychological difficulties among college students [55,56]. Moderate to severe symptoms of insomnia, anxiety, and depression were reported by 26%, 38%, and 32% of our sample, respectively. Previous reports on college students in Italy during the COVID-19 pandemic indicated a worsening of sleep and psychological health [57,58], suggesting that we captured in this sample a still-lasting effect of enduring isolation due to COVID-19 safety rules and lack of social contacts. In particular, the average ISI score was above threshold for clinical insomnia, slightly higher than a previous report in a comparable sample (9.3 ± 6.23) [57], and lower than scores found in another similar sample (13.8 ± 3.3) [58]. Consistent with previous literature [59,60], the stronger predictors of insomnia severity were poor sleep hygiene, anxiety, and depressive symptoms. Good sleep hygiene is often challenged in college students by social norms, lifestyle changes, and new stressors (e.g., exams), increasing the likelihood of displaying maladaptive sleep habits, such as irregular sleep–wake schedules and use of the bed for multiple activities [59]. The new challenges faced in the transition to university also increase levels of anxiety and depression [61], which are closely associated with insomnia symptoms [60]. Unhelpful pre-sleep behaviours can concur with psychological factors in deteriorating sleep in college students. In our sample, avoidant coping was also associated with higher insomnia symptoms. Avoidant coping is characterised by attempts to suppress unwanted thoughts in the face of stressful events; strategies include the use of distractions or denial [62]. This type of coping strategy has been associated with insomnia symptoms, probably due to increased physiological arousal [63]. Future studies should further investigate the association between coping style and insomnia in young populations.

Although we found mostly small correlations between strategies used to facilitate sleep onset and insomnia symptoms, it is worth noting that some behaviours were positively associated with insomnia (e.g., listening to music) but not others (e.g., using electronic devices). This unexpected result needs to be replicated; however, it indicates the need for in-depth investigations of the sleep-onset facilitators used by those with higher insomnia symptoms, as the strategies to facilitate sleep onset can differ compared to those with less sleep difficulties. Moreover, while different behaviours were used to facilitate sleep onset, no correlation was negative, indicating that no sleep-onset facilitator was associated with lower insomnia symptoms. The association between pre-sleep behaviours to facilitate sleep onset and insomnia symptoms is likely bidirectional: on one hand, sleep difficulties may increase the employment of sleep-onset facilitators [38]; on the other hand, the use of sleep-onset facilitators can increase sleep difficulties because of their direct harmful effect and by increasing psychophysiological arousal [2]. These mechanisms need further investigation because they can impose a vicious circle between pre-sleep behaviours and insomnia symptoms.

The present study adds to the growing literature underlining the importance of considering motivational factors of pre-sleep behaviours. Indeed, a high proportion of students recurred to common pre-sleep behaviours to facilitate sleep onset. Understanding the motivations behind pre-sleep behaviours can help inform preventive programs for college students. Moreover, the use of substances and medications to aid sleep is of particular concern, as this behaviour can have detrimental health consequences. In our study, the students using behaviours to aid sleep onset were characterised by more psychological difficulties (anxiety) and higher insomnia symptoms. The latter result suggests that, although used to self-medicate sleep-onset problems, the strategies used are not effective in reducing insomnia symptoms. Indeed, there was no negative association between sleep-onset facilitators and insomnia symptoms. Interestingly, a low percentage of students perceived pre-sleep behaviours to be effective in helping sleep. This possible discrepancy and associated mechanisms need to be addressed in future research and considered in intervention programs.

### 3.1. Implications

While pre-sleep behaviours are recognised as a focus of treatment, especially in younger populations [64], there are still gaps in our understanding of how to obtain behavioural changes. Intentions and motivations can be particularly relevant in the choice and maintenance of pre-sleep behaviours.

The evidence that some behaviours are used to facilitate sleep onset suggests that caution should be paid when replacing them. As a coping mechanism against sleep difficulties, removing a behaviour may create even more psychological arousal in the absence of a more positive alternative. Preventive programs aimed at behavioural change could focus on sleep health as a multidimensional construct [50] and on supporting self-management of sleep [65] as an alternative to direct attempts to modify behaviours.

Furthermore, sleep-onset facilitators were seldom considered effective in aiding sleep and were not associated with good sleep. Thus, motivations are maintained against the effective results. This could indicate a lack of knowledge about the factors influencing sleep health or of alternative strategies to cope with sleep difficulties but not a lack of motivation to do so. There is, indeed, an opportunity to fill this gap by delivering psychoeducation that gives skills to cope with sleep difficulties when therapeutic options are not available or achievable. Younger populations, especially college students, have less access to evidence-based psychological treatment for insomnia symptoms, particularly Cognitive-Behavioural Therapy for Insomnia (CBT-I) which is the first-line treatment for insomnia disorder [42]. Digitally delivered CBT-I, which has also been recommended together with face-to-face delivery by European Guidelines [6], could be a more viable approach for chronic sleep difficulties in this population.

Future research could further investigate the motivations behind pre-sleep behaviours, employing prospective designs and gathering more information on specific pre-sleep behaviours across age groups.

### 3.2. Limitations

The present study is not free of limitations. It should be noted that our results are based on self-reported data, which are prone to recall bias. Pre-sleep behaviours were investigated in reference to the previous week to reduce recall bias. This assessment was used as a proxy of a habitual week, considering that habits form over time [66]. However, insomnia symptoms were referred to the previous month to assess symptoms on a longer time scale, thus reducing the probability that symptoms were brief responses to situational stressors. This temporal difference should be considered when interpreting the results. We could not identify the moment in which a specific behaviour became a habit. Future research with a longitudinal design could investigate how the development of a pre-sleep behaviour as a habit is associated with the onset of insomnia symptoms. Moreover, it should also be noted that we did not assess the differences between weekday and weekend behaviours, which can differ based on personal habits. A deeper investigation, also employing multiple measurements (e.g., momentary ecological assessment) and physiological recordings (e.g., actigraphy), is needed to model how pre-sleep behaviours and sleep-aid motivation interacts with sleep patterns across the week. Secondly, our sample was selected based on age range and was recruited through social media groups and pages for students, which could limit its representativeness of the college student population; while the mean age and the percentage of students living with the family of origin were similar to that of the Italian population of college students, the sex imbalance was wider (based on national statistics: https://ustat.mur.gov.it/dati/didattica/italia/atenei) (accessed on 27 June 2024). Furthermore, other sociodemographic variables that can influence sleep (e.g., race, income) were not recorded.

Thirdly, cross-sectional data do not allow for conclusions on causal relationships to be drawn. Future directions include longitudinal data to elucidate the influence of sleep problems in shaping pre-sleep behaviours and vice versa. A last limitation is that we recruited students in a period still influenced by the previous COVID-19 pandemic restrictions. This could partially explain the high level of insomnia symptoms found in the sample, in accordance with evidence of sleep deterioration after COVID-19 [57]. The students in our study had prevalently good sleep hygiene, which is not common in college students. Our sample also had a higher prevalence of evening chronotypes, which can be reflective of the relatively young age (as eveningness is more prevalent during adolescence and early adulthood [67]) and could have influenced evening activities. Our sample was also young and mostly composed of females. Thus, cautions should be paid in generalising our results to different samples.

## 4. Materials and Methods

### 4.1. Participants and Procedure

Participants were recruited to engage in a study aiming at investigating sleep habits and associated factors in college students. The inclusion criteria were an age between 18 and 30 years and being currently enrolled in any degree program at an Italian University. The questionnaire was developed on Google Forms (Google^TM^), an online platform easily accessible without a need to log in to personal e-mails, and distributed through Facebook groups and social media pages of Student Associations. Both channels gather university students seeking academic information and involvement in the students’ community. Given that voluntary participation in psychological research is linked to personal interests [68], we expected to reach students with sleep difficulties or poor satisfaction for sleep who would be more interested in research about sleep. This allows for a clearer understanding of the association between pre-sleep behaviours and sleep and psychological variables in a sample at risk for insomnia disorder. The invitation consisted of a short description of the study and the link to complete the online survey. To continue with the survey, participants were asked to provide online informed consent. Participants did not receive any form of compensation. The survey took approximately 15 min to complete and was accessible online from 6 April to 31 July 2022. This study was performed in accordance with the 1964 Helsinki Declaration and later amendments and approved by the Ethical Committee of Guglielmo Marconi University of Rome (Approval: 2 March 2022).

### 4.2. Assessment

Demographic data were collected, including age, sex, working status, living situation, and marital status. Academic information included current year grade point average (GPA), the region in which they attend university, and their college year and area of study. In order to better characterise the sample, the students were asked to indicate when their next exam was due, choosing between the following options: (a) “within 7 days”; (b) “between 7 and 14 days”; (c) “between 14 and 21 days”; (d) “in more than 21 days”; (e) “I have completed all the exams of my course”. This variable was used to characterise the sample in terms of exam proximity, considered a stressful situation that may impact sleep.

Participants completed the following questionnaires:

- Insomnia Severity Index (ISI) [69]: Participants rated their level of insomnia symptoms in the past month. The ISI is a validated measure of 7 items on a five-point Likert scale. A higher total score indicates worse insomnia symptoms (range 0 to 28). The total score is interpreted as follows: clinically irrelevant insomnia (0–7); subthreshold insomnia (8–14); moderate insomnia (15–21); and severe insomnia (22–28). Internal consistency of the Italian version is good (α = 0.75) [70]; Cronbach’s alpha in our sample was 0.83.
- Hospital Anxiety and Depression Scale (HADS) [71]: The questionnaire consists of 7 items rating anxiety and 7 items rating depressive symptoms during the preceding week. Each item is scored from 0 to 3. Based on the individual sum scores, participants are defined as non-case (0–7); mild (8–10); moderate (11–14); or severe (15–21). Cronbach’s alpha of the Italian version is 0.89 [72]; in our sample, Cronbach’s alpha was 0.80.
- Sleep Hygiene Index (SHI) [25,73]: The SHI is a 13-item questionnaire evaluating sleep hygiene practices. Participants rated how frequently they engage in specific behaviours during the past week on a scale from 1 = never to 5 = always. Higher scores are indicative of poorer sleep hygiene. The total score can be interpreted as follows: good sleep hygiene habits (≤26); average (27–34); and poor (>34) [25]. The Italian version of the SHI demonstrated good psychometric properties, with a good internal consistency (Cronbach’s alpha 0.74) [73]; in our sample, internal consistency was acceptable (α = 0.66).
- Morningness–Eveningness Questionnaire Reduced (MEQr) [74]: The MEQr is a short and validated measure of circadian typology. Higher total scores indicate more morning preference. Chronotype is defined by cut-off scores: evening-type (<11); intermediate-type (11–18); and morning-type (>18). In a similar sample (age 18–30 years), the Cronbach’s alpha was 0.71 [75]; in our sample, Cronbach’s alpha was 0.68.
- Brief-COPE [76]: A 28-item questionnaire measuring coping strategies. Participants were asked to rate how frequently they use each coping strategy from 1 = “I haven’t been doing this at all” to 4 = “I’ve been doing this a lot”. Average scores are obtained for three overarching coping styles: problem-focused, emotion-focused, and avoidant coping. The internal consistency of the Italian version was found to be good (α = 0.81) [77]; in the current study, internal consistency was also good (α = 0.72).
- Sleep Patterns: Four questions were developed to measure sleep patterns during weekdays and weekends. The questions were presented with reference to the typical week of a period with low exam pressure (more than 21 days before an examination) and of a period with high exam pressure (the week before an examination). Participants were asked to choose, for each period, the response that better represents the hours in which they go to bed and wake up during the week and the weekend. This variable was recorded for descriptive purpose.
- Frequency of Pre-Sleep Behaviours: Students were presented with a list of 22 common behaviours and asked to rate how many days in the previous week (from 0 to 7) they had performed each behaviour the hour before going to bed. Pre-sleep behaviours were coded as consistent bedtime routines when implemented at least three days per week, following the definition applied to young children [3]. These behaviours included doing yoga, meditation, taking a hot shower or bath, drinking herbal tea, drinking alcohol, smoking cigarettes, smoking cannabis, using other substances, taking prescribed medications, taking non-prescribed medications, taking melatonin, taking an antihistamine, spending time on social media, watching TV shows, drinking coffee, drinking tea, drinking soft drinks, drinking energy drinks, drinking hot cocoa, reading, listening to music, and doing homework. These activities were chosen based on previous literature of common pre-sleep behaviours in adults [36,78,79] and children [3].
- Use of behaviours as sleep-onset facilitators: A list of pre-sleep behaviours was presented to students, and they were asked to choose all the behaviours they had enacted to help them fall asleep more easily/rapidly (here referred to as “sleep-onset facilitators”). They could choose more than one behaviour. For each behaviour, the responses were coded as 1 (behaviour selected as a sleep-onset facilitator) and 0 (behaviour not selected as a sleep-onset facilitator).
- Perceived effectiveness of behaviours in aiding sleep onset: To record the perceived positive effect of pre-sleep behaviours on sleep onset, the next question asked students to select the behaviours they perceived to be effective in promoting sleep onset, regardless of motivations (here referred to as “effective in aiding sleep onset”). The response to this question was coded as 1 (behaviour selected as effective in aiding sleep onset) and 0 (behaviour not selected as effective in aiding sleep onset). This variable was assessed for descriptive purposes to gain insight into the strategies considered more or less effective in facilitating sleep onset.

### 4.3. Statistical Analysis

The collected data were analysed using SAS Software 9.4 version with SAS/STAT version 14.1 (SAS Institute Inc., Cary, NC, USA) by a professional biostatistician (S.C.).

Descriptive statistics were performed on the study variables. Specific pre-sleep behaviours and strategies to facilitate sleep onset were described as means (M) and standard deviations (SD) and ranges and/or in absolute and relative frequencies, depending on the type of variable. Because each student could report more than one sleep-onset facilitator, we did not compare groups of behaviours; instead, we performed independent sample *t*-tests with the Satterthwaite method on each behaviour, comparing the students reporting use as a sleep-onset facilitator and those not reporting this motivation on insomnia symptoms (ISI), sleep hygiene (SHI), anxiety and depression (HADS), and avoidant coping (BRIEF-Cope). These analyses were performed for descriptive purpose in accordance with the aim to characterise the students using behaviours as sleep-onset facilitators and students not reporting this motivation. We did not aim to describe the effect of sleep-onset facilitators on sleep above covariations; consequently, we did not consider covariates in *t*-test analysis. For correction, we used the Satterthwaite’s method, which is one of the Student’s *t*-test correction methods suitable for cases of small sample size. With this method, the test correction on distributions with unequal variances is performed on the number of degrees of freedom, which is always smaller than the classical ones for the Student’s *t*-test with two independent samples, and in a more marked way, the greater the difference between the two variances. It follows that the test is more conservative, that is, it is more difficult to reject the null hypothesis, which is the Type I error that different variances tend to increase.

Correlation analysis was performed on all variables. A multivariable linear regression model was used to determine the association between severity of insomnia symptoms and multiple independent variables, including all pre-sleep behaviours assessed in the sample and known predictors of insomnia (depression and anxiety, sleep hygiene, chronotype), demographic variables (age, sex), and factors that can be associated with higher insomnia symptoms (exam proximity and coping strategies). Using the G*Power Software (version 3.1.9.6) [80], the required sample size to achieve adequate power (medium effect size, power = 0.80, α = 0.05) for the multivariable linear regression with 33 predictors was 195. The stepwise method was used to determine the best fitting model and which pre-sleep behaviours (performed independently from motivation) contribute to insomnia symptoms. To test our hypothesis that the use of behaviours as sleep-onset facilitators would be associated with insomnia symptoms, we performed Spearman’s rank correlation analysis between pre-sleep behaviours as sleep-onset facilitators and (a) ISI total score and (b) the score of the first item of the ISI, which specifically assesses the severity of difficulties in initiating sleep.

For all analyses, statistical significance was set at *p <* 0.05.

## 5. Conclusions

The current findings offer a comprehensive assessment of pre-sleep behaviours, bedtime routines, and strategies to facilitate sleep onset in young college students. The present study adds to the growing literature on the motivations sustaining pre-sleep behaviours and their association with sleep and psychological health. Our results indicate that a high percentage of young college students display bedtime behaviours considered incongruent with sleep and employ different methods to aid sleep onset, which are rarely perceived as effective. How this mechanism maintains over time and how it is associated with overall sleep health need to be further investigated. A deeper understanding of pre-sleep behaviours in young adults may inform on preventive and intervention programs for sleep problems aimed at behavioural changes.

## Figures and Tables

**Table 1:** Demographic, sleep, and psychological variables in the total sample.

	Total (*n* = 548)			
Demographic Variables	**N**	**%**	**Mean (SD)**	**Range**
Age			23.48 (2.77)	19–30
19–24	360	65.7		
25–30	188	34.3		
Sex				
Male	107	19.5		
Female	441	80.5		
Pscyhological and sleep variables	**N**	**%**	**Mean (SD)**	**Range**
Insomnia symptoms (ISI)			10.52 (5.81)	0–27
Absence of insomnia	190	34.7		
Subthreshold insomnia	215	39.2		
Moderate insomnia	118	21.5		
Severe insomnia	25	4.6		
Anxiety symptoms (HADS-A)			9.23 (4.00)	0–18
non-case	189	34.5		
mild	150	27.4		
moderate	174	31.8		
severe	35	6.4		
Depressive symptoms (HADS-D)			8.29 (3.97)	0–21
non-case	245	44.7		
mild	129	23.5		
moderate	145	26.5		
severe	29	5.3		
Sleep hygiene (SHI)			18.52 (7.30)	0–42
Good	468	85.4		
Average	68	12.4		
Poor	12	2.2		
Circadian typology (MEQr)			13.65 (3.97)	4–23
Evening	120	21.9		
Intermediate	362	66.1		
Morning	66	12.0		

**Table 2:** Prevalence of reported pre-sleep behaviours, use as sleep-onset facilitators, and perceived effectiveness in aiding sleep.

	Students Reporting the Pre-Behaviour at Least Once a Week		Use to Facilitate Sleep Onset				Prevalence of Use as Sleep-Onset Facilitators in the Sample	Prevalence of Perceived Efficacy in Aiding Sleep
	N	%	No		Yes		%	%
			N	%	N	%		
Spending time on social media	538	98.2	298	55.4	240	44.6	43.8	15.0
Watching TV shows on any device	475	86.7	194	40.8	281	59.2	51.3	29.9
Taking a hot bath or shower	338	61.7	226	66.9	112	33.1	20.4	14.6
Listening to music, radio, podcast	302	55.1	169	56.0	133	44.0	24.3	15.3
Reading	275	50.2	177	64.4	98	35.6	17.9	14.1
Doing homework	236	43.1	176	74.6	60	25.4	10.9	3.8
Drinking herbal tea	226	41.2	145	64.2	81	35.8	14.8	11.3
Drinking alcohol	216	39.4	185	85.6	31	14.4	5.7	3.1
Smoking cigarettes	170	31.0	118	69.4	52	30.6	9.5	1.5
Drinking soft drinks	137	25.0	131	95.6	6	4.4	1.1	0.0
Taking melatonin	122	22.3	43	35.2	79	64.8	14.4	12.6
Drinking tea	88	16.1	75	85.2	13	14.8	2.4	0.5
Drinking coffee	88	16.1	84	95.5	4	4.5	0.7	1.1
Taking antihistamine	68	12.4	58	85.3	10	14.7	1.8	1.3
Doing meditation	66	12.0	45	68.2	21	31.8	3.8	4.6
Taking cannabis	55	10.0	35	63.6	20	36.4	3.6	5.1
Drinking hot cocoa	52	9.5	47	90.4	5	9.6	0.9	0.2
Taking prescribed sleep medications	44	8.0	16	36.4	28	63.6	5.1	3.5
Doing yoga	36	6.6	29	80.6	7	19.4	1.3	2.0
Taking non-prescribed sleep medications	32	5.8	19	59.4	13	40.6	2.4	1.3
Drinking energy drinks	28	5.1	24	85.7	4	14.3	0.7	0.0
Using other substances	14	2.6	10	71.4	4	28.6	0.7	0.7

**Table 3:** Group comparison (independent *t*-test) between students employing pre-sleep behaviours as sleep-onset facilitators and students employing behaviours for other motivations.

Relaxing Behaviours			
Variable	Use as Sleep-Onset Facilitator	Use for Other Motivation	Group Comparison
	*n* = 303	*n* = 188	
	M (SD)	M (SD)	*t*-Test
Insomnia (ISI)	11.39 (5.77)	9.42 (5.84)	**3.65**
Chronotype (MEQr)	13.33 (4.07)	14.25 (3.81)	**2.53**
Sleep Hygiene (SHI)	18.97 (7.48)	18.21 (7.18)	*ns*
Anxiety (HADS-A)	9.54 (3.94)	9.07 (4.06)	*ns*
Depression (HADS-D)	8.22 (4.05)	8.28 (3.91)	*ns*
Problem-Focused Coping (BRIEF-COPE)	2.68 (0.57)	2.72 (0.51)	*ns*
Emotion-Focused Coping (BRIEF-COPE)	2.38 (0.39)	2.3 (0.38)	**2.41**
Avoidant Coping (BRIEF-COPE)	1.82 (0.44)	1.72 (0.39)	**2.76**
**Substance Use**			
**Variable**	**Use as Sleep-Onset Facilitator**	**Use for Other Motivation**	**Group Comparison**
	***n* = 84**	***n* = 203**	
	**M (SD)**	**M (SD)**	***t*** **-Test**
Insomnia (ISI)	13.26 (5.86)	10.49 (5.52)	**3.7**
Chronotype (MEQr)	12.58 (3.91)	13.73 (3.82)	**2.28**
Sleep Hygiene (SHI)	22.68 (7.74)	19.23 (6.93)	**3.54**
Anxiety (HADS-A)	10.25 (4.06)	9.15 (3.81)	**2.13**
Depression (HADS-D)	8.89 (3.66)	8.01 (3.61)	*ns*
Problem-Focused Coping (BRIEF-COPE)	2.73 (0.54)	2.72 (0.50)	*ns*
Emotion-Focused Coping (BRIEF-COPE)	2.40 (0.40)	2.35 (0.38)	*ns*
Avoidant Coping (BRIEF-COPE)	1.97 (0.51)	1.73 (0.38)	**3.9**
**Medication Use**			
**Variable**	**Use as Sleep-Onset Facilitator**	**Use for Other Motivation**	**Group Comparison**
	***n* = 114**	***n* = 83**	
	**M (SD)**	**M (SD)**	***t*** **-Test**
Insomnia (ISI)	14.12 (5.31)	10.52 (5.69)	**4.51**
Chronotype (MEQr)	12.23 (4.11)	14.63 (4.06)	**4.07**
Sleep Hygiene (SHI)	20.81 (7.66)	18.17 (7.50)	**2.42**
Anxiety (HADS-A)	10.68 (3.85)	9.10 (4.05)	**2.77**
Depression (HADS-D)	9.04 (3.83)	7.96 (4.08)	*ns*
Problem-Focused Coping (BRIEF-COPE)	2.69 (0.57)	2.76 (0.53)	*ns*
Emotion-Focused Coping (BRIEF-COPE)	2.43 (0.38)	2.35 (0.44)	*ns*
Avoidant Coping (BRIEF-COPE)	1.94 (0.48)	1.74 (0.42)	**3.04**
**Electronic Devices**			
**Variable**	**Use as Sleep-Onset Facilitator**	**Use for Other Motivation**	**Group Comparison**
	***n* = 356**	***n* = 187**	
	**M (SD)**	**M (SD)**	***t*** **-Test**
Insomnia (ISI)	10.54 (5.83)	10.50 (5.75)	*ns*
Chronotype (MEQr)	13.67 (3.91)	13.60 (4.09)	*ns*
Sleep Hygiene (SHI)	18.83 (7.17)	17.99 (7.53)	*ns*
Anxiety (HADS-A)	9.40 (4.00)	8.87 (3.97)	*ns*
Depression (HADS-D)	8.27 (3.96)	8.32 (4.00)	*ns*
Problem-Focused Coping (BRIEF-COPE)	2.71 (0.54)	2.69 (0.55)	*ns*
Emotion-Focused Coping (BRIEF-COPE)	2.37 (0.39)	2.32 (0.37)	*ns*
Avoidant Coping (BRIEF-COPE)	1.80 (0.44)	1.71 (0.37)	**2.56**

Note: *t*-test results are reported only for significant comparisons (*p* < 0.05) in bold. *ns* = not significant.

**Table 4:** Correlation between psychological variables, sleep variables, and frequency of pre-sleep behaviours.

Psychological and Sleep Variables								
	Problem-Focused Coping	Emotion-Focused Coping	Avoidant Coping	ISI	rMEQ	SHI	HADS-A	HADS-D
Problem-Focused Coping	1	0.41 *	−0.13 *	−0.11 *	0.12 *	−0.08 *	−0.15 *	−0.28 *
Emotion-Focused Coping		1	0.26 *	0.09 *	−0.03	0.15 *	0.16 *	0.02
Avoidant Coping			1	0.28 *	−0.22 *	0.32 *	0.24 *	0.17 *
ISI				1	−0.23 *	0.46 *	0.45 *	0.41 *
rMEQ					1	−0.42 *	−0.11 *	−0.14 *
SHI						1	0.36 *	0.28 *
HADS-A							1	0.59 *
HADS-D								1
**Pre-Sleep Behaviours and Psychological and Sleep Variables**								
	**Problem-Focused Coping**	**Emotion-Focused Coping**	**Avoidant Coping**	**ISI**	**rMEQ**	**SHI**	**HADS-A**	**HADS-D**
Yoga	0.11 *	0.13 *	0.08	0.05	0.00	0.00	0.02	−0.02
Meditation	0.07	0.19 *	0.11 *	0.08	−0.01	0.01	0.04	0.03
Shower/Bath	0.04	0.03	0.06	0.07	−0.02	0.07	0.07	−0.02
Herbal tea	0.07	0.07	0.10 *	0.11 *	−0.04	0.04	0.04	0.01
Alcohol	0.00	0.03	0.16 *	0.10 *	−0.10 *	0.20 *	0.02	−0.02
Smoking	0.06	0.06	0.08	0.09 *	−0.11 *	0.24 *	0.05	0.01
Cannabis	0.03	0.08	0.14 *	0.04	−0.08	0.16 *	0.03	−0.01
Other substances	0.03	0.10 *	0.18 *	0.11 *	−0.04	0.17 *	0.13 *	0.06
Meds Prescribed	−0.06	0.07	0.21 *	0.23 *	−0.07	0.11 *	0.13 *	0.13 *
Meds Non-prescribed	−0.06	0.03	0.12 *	0.16 *	−0.11 *	0.06	0.11 *	0.07
Melatonin	0.06	0.08	0.09 *	0.27 *	−0.11 *	0.16 *	0.14 *	0.03
Antihistamine	0.08	0.04	0.04	0.06	−0.02	−0.01	−0.04	−0.05
Social media	0.03	0.08	−0.01	−0.01	−0.05	0.21 *	0.03	0.00
TV Shows	0.10 *	0.03	0.07	0.01	−0.01	0.17 *	0.02	−0.08
Coffee	0.10 *	0.12 *	0.08	0.01	−0.01	0.11 *	0.07	0.03
Tea	−0.03	0.00	0.05	0.04	−0.02	0.04	0.06	0.05
Soft drinks	0.05	0.01	0.07	0.07	−0.07	0.14 *	0.07	0.07
Energy drinks	−0.01	0.09 *	0.11 *	0.03	−0.01	0.06	0.11 *	0.03
Hot cocoa	0.06	0.03	−0.02	−0.01	0.02	0.06	0.04	0.00
Reading	0.05	0.11 *	0.17 *	0.03	0.04	0.06	0.00	−0.13 *
Music	−0.01	0.09 *	0.20 *	0.09 *	−0.12 *	0.17 *	0.04	0.01
Homework	0.09 *	0.11 *	0.10 *	0.12 *	−0.07	0.12 *	0.18 *	0.08

Note: * Significant correlations (*p* < 0.05) in bold.

**Table 5:** Result of stepwise linear regression model.

Stepwise Selection: Step 12					
R-Square = 0.4099 and C(p) = 4.2336					
Analysis of Variance					
**Source**	**DF**	**Sum of** **Squares**	**Mean** **Square**	**F Value**	**Pr > F**
Model	12	7577.561	631.4634	30.96	<0.0001
Error	535	10,911	20.39445		
Corrected Total	547	18,489			
**Variable**	**Parameter** **Estimate**	**Standard** **Error**	**Type II SS**	**F Value**	**Pr > F**
Intercept	5.16348	2.05273	129.0431	6.33	0.0122
Age	−0.14352	0.07299	78.84354	3.87	0.0498
Uni_ExamsProximity	−0.28216	0.14905	73.09046	3.58	0.0589
Coping_Avoidant	1.01668	0.50692	82.03385	4.02	0.0454
SHI_TOT	0.24731	0.03046	1344.554	65.93	<0.0001
HADS_Anx_TOT	0.23184	0.06306	275.6337	13.52	0.0003
HADS_Dep_TOT	0.28478	0.06098	444.8032	21.81	<0.0001
PSB_Meditation	0.28881	0.19844	43.19816	2.12	0.1462
PSB_MedsPrescribed	0.45347	0.15267	179.9227	8.82	0.0031
PSB_Melatonin	0.54345	0.09951	608.2418	29.82	<0.0001
PSB_Antihistamine	0.21817	0.1249	62.22702	3.05	0.0813
PSB_Social media	−0.36957	0.11844	198.5796	9.74	0.0019
PSB_Hot Cocoa	−0.45343	0.21868	87.68104	4.3	0.0386

Note: Uni_examsProximity: proximity to next exam (coded as 1 = Within 7 days; 2 = Within 7–14 days; 3 = Within 14–21 days; 4 = After 21 days; 5 = No pending exam). Coping_Avoidant: avoidant coping assessed with the Brief-COPE. SHI = Sleep Hygiene Index; HADS_Anx = Anxiety subscale of the Hospital Anxiety and Depression Scale; HADS_Dep = Depression subscale of the Hospital Anxiety and Depression Scale; PSB = Pre-sleep behaviour.

**Table 6:** Correlation between pre-sleep behaviours used as sleep-onset facilitators and insomnia symptoms.

	Insomnia Symptoms (ISI) *n* = 548	Sleep-Onset Difficulties (ISI Item 1) *n* = 548
Use as Sleep-Onset Facilitator	Spearman’s Rho	Spearman’s Rho
Meditation	0.08	0.04
Hot bath or shower	0.02	0.07
Herbal tea	0.11 *	0.11 *
Alcohol	0.14 *	0.04
Cigarette smoking	0.17 *	0.19 *
Cannabis	0.05	0.04
Other substances	0.08	0.02
Prescribed medications	0.19 *	0.16 *
Non-prescribed medications	0.10 *	0.08
Melatonin	0.26 *	0.19 *
Antihistamine	0.12 *	0.11 *
Social	0.00	0.04
TV shows	0.03	0.04
Coffee	−0.01	0.00
Tea	0.01	0.06
Soft drinks	0.08	0.06
Energy drinks	0.04	0.07
Hot cocoa	0.03	0.03
Reading	0.09 *	0.03
Music	0.14 *	0.15 *
Doing homework	0.09 *	0.05

Note: * Significant correlations (*p* < 0.05) in bold. ISI = Insomnia Severity Index. ISI item 1 assesses sleep-onset difficulties.

## Data Availability

The data presented in this study are available on request from the corresponding author due to privacy or ethical restrictions.

## Supplementary Materials

The following supporting information can be downloaded at: https://www.mdpi.com/article/10.3390/clockssleep6030031/s1, Figure S1: Forest plot for stepwise linear multivariable regression model. Uni_examsProximity: proximity to next exam (coded as: 1 = Within 7 days; 2 = Within 7–14 days; 3 = Within 14–21 days; 4 = After 21 days; 5 = No pending exam). Coping_Avoidant: avoidant coping assessed with the Brief-COPE. SHI= Sleep Hygiene Index; HADS_Anx = Anxiety subscale of the Hospital Anxiety and Depression Scale; HADS_Dep = Depression subscale of the Hospital Anxiety and Depression Scale; PSB = Pre-sleep behaviour; Table S1: Sociodemographic and Academic variables of the sample (*n* = 548); Table S2: Sleep patterns of students in weekdays and weekend in a recent exam period (the week before an exam) and non-exam period (more than 3 weeks before an exam); Table S3: Frequency of pre-sleep behaviours in a 7-day period.
