# Supplementary material for: A Comprehensive Assessment of Bedtime Routines and Strategies to Aid Sleep Onset in College Students: A Web-Based Survey
Source: Clocks Sleep. 2024 Aug 29;6(3):468–87. doi: 10.3390/clockssleep6030031 (PMC11417809; doi:10.3390/clockssleep6030031)
Supplement: Supplementary file 1 [file clockssleep-06-00031-s001.zip › clockssleep-3027431-supplementary.pdf]

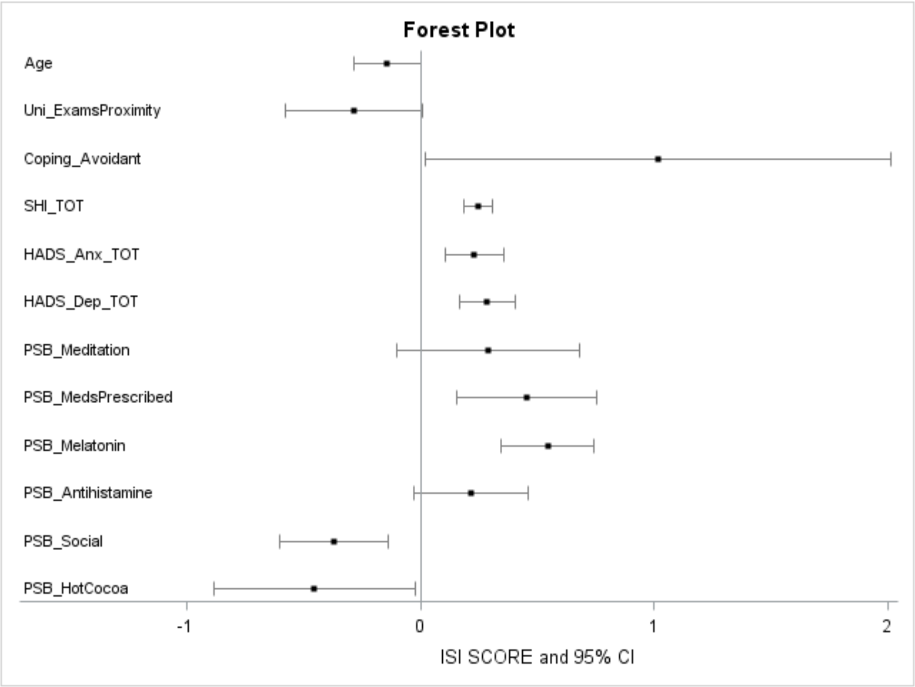

Supplementary Figure S1. Forest plot for stepwise linear multivariable regression model. Uni_examsProximity: proximity to next exam (coded as: 1= Within 7 days; 2= Within 7-14 days; 3= Within 14-21 days; 4= After 21 days; 5= No pending exam). Coping_Avoidant: avoidant coping assessed with the Brief-COPE. SHI= Sleep Hygiene Index; HADS_Anx= Anxiety subscale of the Hospital Anxiety and Depression Scale; HADS_Dep= Depression subscale of the Hospital Anxiety and Depression Scale; PSB= Pre-sleep behaviour.

Supplementary Table S1. Sociodemographic and Academic variables of the sample (n=548).

Sociodemographic and Academic variables	N	%
Occupation		
Student	412	75.2
Student worker	136	24.8
Marital status		
In a relationship	303	55.6
Single	235	43.1
Married	7	1.3
Missing	3	0.5
Living situation		
With roommates	138	23.3
With partner	39	7.1
With family of origin	352	64.6
Alone	16	2.9
Missing	3	0.5
Disciplinary fields		
Medicine	83	15.1
Pedagogy and Psychology*	76	13.9
Industrial and Information Engineering	63	11.5
Antiquities, Philology, Literary Studies, Art History	58	10.6
Economics and Statistics	34	6.2
History and Philosophy	34	6.2
Other Fields**	109	19.9
Not Specified (Other)	91	16.6
Italian Macroregion		
North-West	108	19.7
North-East	68	12.4
Centre	148	27.0
South	196	35.8
Islands	28	5.1
GPA		

1.0	8	1.46
2.0	48	8.76
3.0	233	42.52
4.0	259	47.26
Days until next exam		
Within 7 days	139	25.4
Within 7 – 14 days	120	21.9
Within 14 – 21 days	95	17.3
After 21 days	138	25.2
All exams done	56	10.2

Note*: The Italian university system includes pedagogy and psychology in the same disciplinary field of history and philosophy. Here they are dived for better description of the sample. ***Other fields* includes: Agricultural and Veterinary Sciences; Law Studies; Mathematics and Informatics; Chemistry; Earth Sciences; Physics; Political and Social Sciences; Civil Engineering and Architecture; Biology. Italian Macroregions (first-level NUTS of the European Union) of attended university are composed as follows: North-West includes Aosta Valley, Liguria, Lombardy, Piedmont; North-East includes Emilia-Romagna, Friuli-Venezia Giulia, Trentino-South Tyrol, Veneto; Centre includes Lazio, Marche, Tuscany, Umbria; South includes Abruzzo, Apulia, Basilicata, Calabria, Campania, Molise; Islands include Sardinia and Sicily. GPA: Grade Point Average; conversion was made from the Italian university system (marks from 18 to 30) to the GPA system.

Supplementary Table S2. Sleep patterns of students in weekdays and weekend in a recent exam period (the week before an exam) and non-exam period (more than 3 weeks before an exam).

**Non-exam period**			**Exam period**			
What time, usually, do you go to sleep during the days of the week?			***What time, usually, do you go to sleep during the days of the week?***			
	**N**	**%**		**N**	**%**	**Diff**
Before 9 pm	0	-	*Before 9 pm*	3	0.55	0
Between 9.01 pm and 10.15 pm	27	4.93	*Between 9.01 pm and 10.15 pm*	67	12.23	7.3
Between 10.16 pm and 0.30 am	287	52.37	*Between 10.16 pm and 0.30 am*	299	54.56	2.19
Between 0.30 am and 2.15 am	202	36.86	*Between 0.30 am and 2.15 am*	152	27.74	-9.12
After 2.16 am	32	5.84	*After 2.16 am*	27	4.93	-0.91
What time do you usually wake up during the days of the week?			***What time do you usually wake up during the days of the week?***			
	**N**	**%**		**N**	**%**	**Diff**
Before 6.30 am	30	5.47	*Before 6.30 am*	34	6.2	0.73
Between 6.31 and 7.45 am	120	21.9	*Between 6.31 and 7.45 am*	196	35.77	13.87
Between 7.46 and 9.45 am	290	52.92	*Between 7.46 and 9.45 am*	260	47.45	-5.47
Between 9.46 and 10.45 am	74	13.5	*Between 9.46 and 10.45 am*	45	8.21	-5.29
After 10.46 am	34	6.2	*After 10.46 am*	13	2.37	-3.83
What time do you usually go to sleep on the weekend?			***What time do you usually go to sleep on weekends?***			-
	**N**	**%**		**N**	**%**	**Diff**
Before 9 pm	0	-	*Before 9 pm*	0	-	-
Between 9.01 pm and 10.15 pm	5	0.91	*Between 9.01 pm and 10.15 pm*	25	4.56	3.65
Between 10.16 pm and 0.30 am	113	20.62	*Between 10.16 pm and 0.30 am*	189	34.49	13.87

Between 0.30 am and 2.15 am	287	52.37	*Between 0.30 am and 2.15 am*	247	45.07	-7.3
After 2.16 am	143	26.09	*After 2.16 am*	87	15.88	-10.21
What time do you usually wake up during the weekend?			***What time do you usually wake up on weekends?***			-
	**N**	**%**		**N**	**%**	**Diff**
Before 6.30 am	7	1.28	*Before 6.30 am*	10	1.82	0.54
Between 6.31 and 7.45 am	28	5.11	*Between 6.31 and 7.45 am*	83	15.15	10.04
Between 7.46 and 9.45 am	230	41.97	*Between 7.46 and 9.45 am*	276	50.36	8.39
Between 9.46 and 10.45 am	172	31.39	*Between 9.46 and 10.45 am*	124	22.63	-8.76
After 10.46 am	111	20.26	*After 10.46 am*	55	10.04	-10.22
All (*n*=)	548	100.00	All (*n* =)	548	100.00	

Supplementary Table S3. Frequency of pre-sleep behaviours in a 7-day period.

Pre-Sleep Behaviours	Frequency in last week (days) as mean (SD)	Frequency of class intervals (days) as N (%)			
		0	1-2	3-4	5-7
Social	5.75 (1.71)	10 (1.8)	21 (3.8)	87 (15.9)	430 (78.5)
TV shows	4.36 (2.42)	73 (13.3)	56 (10.2)	112 (20.4)	307 (56.0)
Bath	2.13 (2.2)	210 (38.3)	115 (21.0)	133 (24.3)	90 (16.4)
Music	2.13 (2.36)	246 (44.9)	85 (15.5)	108 (19.7)	109 (19.9)
Reading	1.71 (2.14)	273 (49.8)	104 (19.0)	92 (16.8)	79 (14.4)
Homework	1.63 (2.25)	312 (56.9)	75 (13.7)	77 (14.1)	84 (15.3)
Herbal tea	1.46 (2.1)	322 (58.8)	79 (14.4)	79 (14.4)	68 (12.4)
Alcohol	1.00 (1.56)	332 (60.6)	124 (22.6)	70 (12.8)	22 (4.0)
Smoking	1.46 (2.48)	378 (69.0)	34 (6.2)	40 (7.3)	96 (17.5)
Soft drinks	0.63 (1.34)	411 (75.0)	78 (14.2)	45 (8.2)	14 (2.6)
Melatonin	0.95 (2.05)	426 (77.7)	34 (6.2)	33 (6.0)	55 (10.0)
Coffee	0.47 (1.33)	460 (83.9)	48 (8.8)	21 (3.8)	19 (3.5)
Tea	0.51 (1.35)	460 (83.9)	33 (6.0)	35 (6.4)	20 (3.6)
Antihistamine	0.51 (1.58)	480 (87.6)	24 (4.4)	14 (2.6)	30 (5.5)
Meditation	0.30 (0.99)	482 (88.0)	42 (7.7)	17 (3.1)	7 (1.3)
Cannabis	0.29 (1.12)	493 (90.0)	33 (6.0)	9 (1.6)	13 (2.4)
Hot cocoa	0.24 (0.9)	496 (90.5)	28 (5.1)	17 (3.1)	7 (1.3)
Prescribed sleep medications	0.34 (1.34)	504 (92.0)	15 (2.7)	9 (1.6)	20 (3.6)
Yoga	0.14 (0.6)	512 (93.4)	26 (4.7)	8 (1.5)	2 (0.4)
Non prescribed sleep medications	0.20 (0.98)	516 (94.2)	14 (2.6)	8 (1.5)	10 (1.8)
Energy drinks	0.13 (0.7)	520 (94.9)	17 (3.1)	6 (1.1)	5 (0.9)
Other substances	0.08 (0.6)	534 (97.4)	7 (1.3)	4 (0.7)	3 (0.5)
